# Tension regulates the cartilage phenotypic expression of endplate chondrocytes through the α‐catenin/actin skeleton/Hippo pathway

**DOI:** 10.1111/jcmm.18133

**Published:** 2024-02-08

**Authors:** Min Zhang, Shouliang Xiong, Daokuan Gao, Chen Liu, Liang Xiao

**Affiliations:** ^1^ Department of Orthopedics Yijishan Hospital, The First Affiliated Hospital of Wannan Medical College Wuhu China; ^2^ Department of Spine Surgery Yijishan Hospital, The First Affiliated Hospital of Wannan Medical College Wuhu China

**Keywords:** actin, endplate chondrocytes, Hippo pathway, tension, α‐Catenin

## Abstract

The study aimed to investigate the regulatory mechanism of intracellular tension signaling in endplate chondrocytes and its impact on extracellular matrix synthesis. Human endplate chondrocytes were subjected to tension load using Flexcell FX‐5000™, and changes in phenotype, morphology, and the expression of Hippo signaling pathway and α‐Catenin were assessed through various techniques. Through the overexpression of YAP and inhibition of α‐Catenin, the study clarified the intracellular tension signaling pathway and its regulation of extracellular matrix synthesis in endplate cartilage. In vitro‐cultured human endplate chondrocytes significantly suppressed phenotype‐related genes and proteins, accompanied by distinct changes in cytoskeleton morphology. Tension activation resulted in the substantial activation of the Hippo pathway, increased phosphorylation of YAP, and reduced nuclear translocation of YAP. YAP overexpression alleviated the inhibitory effect of tension on extracellular matrix synthesis in endplate chondrocytes. Tension also upregulated the expression of α‐Catenin in endplate chondrocytes, which was attenuated by inhibiting α‐Catenin expression, thereby reducing the impact of tension on cytoskeletal morphology and YAP nuclear translocation. Taken together, the α‐Catenin/actin skeleton/Hippo‐coupled network is a crucial signaling pathway for tension signaling in endplate chondrocytes, providing potential therapeutic targets for the treatment of endplate cartilage degeneration.

## 1 INTRODUCTION

Endplate cartilage is a layer of hyaline cartilage located on the surface of the upper and lower edges of the spinal centrum. It is not only closely related to the longitudinal growth of adjacent centrum but also disperses mechanical loads and repairs the nucleus pulposus. It is also the main pathway to supply nutrition to the intervertebral disc.^1^ Endplate cartilage tissue comprises a small number of endplate chondrocytes and a large amount of extracellular matrix. The extracellular matrix mainly comprises type II collagen as well as aggrecan and elastin. Endplate chondrocytes are surrounded by a dense extracellular matrix and cannot migrate freely. In addition, their synthesis capability is low due to the absence of nerves, blood vessels or lymphoid tissue distribution.^2^, ^3^ Under physiological loading conditions, the metabolic activity in the extracellular matrix of endplate cartilage is maintained in dynamic equilibrium.^4^ When endplate cartilage is under excessive load, the secretory function of endplate chondrocytes will be abnormal, resulting in accelerated decomposition of the extracellular matrix, increased expression of type I collagen, decreased expression of type II collagen and aggrecan, calcification and reconstruction of the subchondral bone, loss of the normal diffusion function of endplate cartilage tissue and degeneration of the intervertebral disc.^5^ Therefore, mechanical loading is considered an important factor affecting the degeneration of endplate cartilage. Cells in the human body transmit signals to the outside mainly through proteins embedded in the cell membrane. During this process, some proteins are dragged by adjacent cell membrane proteins or adhere to other matrix proteins, generating tension signals.^6^ Therefore, tension load is closely related to the biological function of the endplate chondrocytes.^7^

Hippo signal transduction is an evolutionarily conserved pathway that negatively regulates the transcriptional activity of its downstream effector molecule yes‐associated protein (YAP) mainly through phosphorylation to limit tissue overgrowth, which is important to maintain cell proliferation and apoptotic homeostasis.^8^ Previous studies have confirmed a close relationship among the Hippo pathway, intracellular mechanical signalling and cytoskeletal morphology.^9^, ^10^, ^11^ Specifically, extracellular forces promote cell‐ECM adhesions via the development of intracellular contractile filamentous actin (F‐actin) structures containing myosin molecules. This is regulated by bidirectional signalling between RHO, ROCK (RHO‐associated kinase) and myosin activity. Contractile F‐actin structures in turn sustain YAP and TAZ nuclear localization and activity through unidentified molecular effectors. In addition, F‐actin also opposes YAP and TAZ phosphorylation through inhibition of the kinases LATS1 (Large tumour suppressor homologue 1) and LATS2, G‐actin, globular actin. The synergistic effect between the F‐actin and the Hippo pathways has been reported to play an important role in disc degeneration. However, how they mediate tension signal transduction in endplate chondrocytes and ultimately affect the secretion and metabolism of the extracellular matrix of chondrocytes remains unclear.^12^

Cadherin, a cell surface adhesion molecule, binds to intracellular α, β and γ catenins to form a cadherin–catenin complex (CCC), which participates in tissue genesis and morphological differentiation and mediates intercellular signal transduction.^13^, ^14^, ^15^ Previous studies have shown that the CCC is a cell tension signal‐sensitive device that can regulate cell function through mechanical signal transduction.^16^ α‐Catenin is a key mechanically sensitive protein in the CCC and is connected to the cytoskeleton of β‐catenin and actin, respectively, as a bridge to transmit mechanical force and regulate intracellular signalling pathways. However, the specific mechanism remains unclear.^17^, ^18^ This study aimed to elucidate the intracellular tension signalling pathway of endplate cartilage and its regulation of extracellular matrix anabolism using cell‐level experiments, providing a new therapeutic idea to alleviate or reverse endplate cartilage degeneration.

## 2 MATERIALS AND METHODS

### 2.1 Collection of human endplate cartilage tissue specimens and primary cell culture

From November 2020 to November 2021, disc specimens collected from patients who had undergone anterior cervical fusion at Yijishan Hospital were rinsed three times with phosphate‐buffered saline (PBS) solution containing 1% penicillin and streptomycin on an ultra‐clean workbench (Table 1). The ligaments, nucleus pulposus and annulus fibrosus tissues were carefully separated and the remaining cartilage tissue of the endplate was cut into 1‐mm^3^‐sized fragments using a sterile blade. Next, 8 mL of 0.25% Trypsin and 0.2% collagenase type II were added to the specimens sequentially, and then the contents were stirred and digested in a shaker at 37°C for 20 min and 6 h, respectively. After digestion, an equal amount of DMED/F12 medium containing 10% fetal bovine serum (FBS) was added to stop digestion. After centrifugation, the filtrate was filtered using a 70‐μm filter, collected, and centrifuged at 1000 r/min for 5 min. The cell precipitate was mixed thoroughly in 3 mL of DMEM/F12 medium using a pipette to make a cell suspension. The cell viability was measured by Trypan blue staining, and the cells were counted using a cell counter. The cell suspension was seeded in a 10‐cm culture dish at a density of 2 × 10^5^/mL in 10 mL of DMEM/F12 medium containing 15% FBS and 1% penicillin–streptomycin solution. The culture dish was incubated at 37°C and 5% CO_2_. Cell growth was observed and photographed under an inverted microscope every day, and the medium was changed every 3–4 days. When the confluency reached 80%–90%, the cells were sub‐passaged and cultured at a ratio of 1:2 or 1:3. Informed consent was obtained from all surgical patients participating in the study.

**TABLE 1:** Patient demographic data.

Parameters	Value
Inclusion time	November 2020–November 2021
Number of patients, *n*	45
Diagnosis	Cervical spondylotic myelopathy
Sex ratio, M:F	24:21
Age, mean (range), years	58.31 ± 7.42 (57–64)
Operation method	ACDF

### 2.2 Cell mechanical loading

The Flexcell FX‐5000TM strain loading system used in this study can exert cyclic tension on cultured cells. This device was connected to a cell loading plate (BioFlexTM six‐well plate, the inner part of plate was a vacuum unit, and the bottom was coated with deformable flexible polystyrene coated with type I collagen to support cell growth) through a computer. The computer can exert tensile force on attached cells by controlling the deformation of the bottom of the BioFlexTM six‐well plate.

The P2 cells were seeded in a BioFlex™ six‐well multidirectional loading plate coated with collagen type I at a density of 2 × 10^5^/mL and were cultured at 37°C and 5% CO_2_. Cell growth was observed under an inverted phase contrast microscope every day. When the cell confluency reached approximately 80%, the Flexcell FX‐5000™ strain loading system was used to exert 0.5 Hz and 12% elongation tension on the cells at 37°C and 5% CO_2_ for 24 h. The control cells were cultured normally without force.

### 2.3 Phalloidin staining

Two millilitres of 0.3% Triton X‐100 were added to each well of the plate, followed by incubation for 10 min. Next, 100 μL of phalloidin staining solution (Gibco, Invitrogen, USA) was added to the samples, followed by incubation for 10 min in the dark at room temperature. Thereafter, 100 μL of 4′,6‐diamidino‐2‐phenylindole (DAPI) was added to the cells, followed by incubation for 5 min in the dark at room temperature. Changes in cytoskeleton F‐actin were observed and photographed by laser confocal microscopy (Leica, Germany).

### 2.4 Gene transduction

The YAP vector plasmid (F 5′‐GGGACCGGTGCCACCATGCTTACCCATACGACGTCCCAG‐3′, R 5′‐CCGGAATTCCTATAACCATGTAAGAAAGCTTTC‐3′) was constructed and virally transfected into endplate chondrocytes. Similarly, specific short interfering RNA against *α‐catenin* (si‐α‐catenin; Santa Cruz Biotechnology, USA) was cloned into a lentiviral vector and was used for virus infection. After virus infection, puromycin (4 μg/mL) was added to the cells for selection, which was completed when all the target cells not treated with infection died under the effect of drugs. Transduction efficiency was monitored by eGFP fluorescence and was typically >90% as determined by flow cytometry. The overexpression and inhibition efficiencies were detected by reverse transcription‐quantitative polymerase chain reaction (RT‐qPCR) and Western blotting. Cells were expanded for subsequent experiments.

### 2.5 Reverse transcription‐quantitative polymerase chain reaction (RT‐qPCR)

Trizol reagent (Invitrogen, USA) was used to extract total RNA from cartilage tissue and cells according to the manufacturer's instructions. The mRNA expression level was determined using the SYBR® Premix Ex TaqTM II kit (Takara, Japan). The reaction conditions for cDNA amplification were as follows: pre‐denaturation at 95°C for 30 s, denaturation at 95°C for 5 s, annealing and extension at 60°C for 25 s and amplification for 50 cycles. Glyceraldehyde 3‐phosphate dehydrogenase (GAPDH) was used as an internal reference, and the results were analysed by the relative quantitative 2^−ΔΔCt^ method. All the experiments were performed in triplicate, and the primer sequences are shown in Table 2.

**TABLE 2:** PCR primer sequences.

Gene	Primer	Primer sequence (5′ → 3′)
ACAN	F	CATTCACCAGTGAGGACCTCGT
ACAN	R	TCACACTGCTCATAGCCTGCTTC
COL2A1	F	TGAGGGCGCGGTAGAGACCC
COL2A1	R	TGCACACAGCTGCCAGCCTC
SOX9	F	ATCTGAAGAAGGAGAGCGAG
SOX9	R	TCAGAAGTCTCCAGAGCTTG
MMP13	F	TGCTGCATTCTCCTTCAGGA
MMP13	R	ATGCATCCAGGGGTCCTGGC
Ctgf	F	GAACAAATGCTGTGCAGGTGA
Ctgf	R	TCCTGGTAGGAATCGGACCTT
Sgcd	F	GACTCTCATCCGCCACTCTG
Sgcd	R	AGGCATCTTTTCCTCCAGCC
Cyr6	F	CTGCGCTAAACAACTCAACGA
Cyr6	R	GCAGATCCCTTTCAGAGCGG
YAP	F	TCAGACAACAACATGGCAGGA
YAP	R	TTCATGGCTGAAGCCGAGTT
α‐catenin	F	AGCTAGCCGCAGAAATGACT
α‐catenin	R	AGCCAAAACATGGGCCTTCT
GAPDH	F	GCTGAGAACGGGAAGCTTGT
GAPDH	R	GACTCCACGACGTACTCAGC

### 2.6 Western blotting

The cells were lysed, and the protein samples were collected. Next, the protein concentration was quantified using the bicinchoninic acid method. The denatured proteins were separated by sodium dodecyl sulfate‐polyacrylamide gel electrophoresis and then transferred to a nitrocellulose membrane. The membrane was blocked with 5% bovine serum albumin (BSA) for 1 h, followed by incubation with the following primary antibodies overnight at 4°C: ACAN (1:100, 1 μg/mL, Abcam, USA), COL2A1 (1:5000, 1 μg/mL, Abcam, USA), SOX9 (1:5000, 0.5 μg/mL, Abcam, Britain), MMP13 (1:3000, 0.5 μg/mL, Abcam, Britain), F‐actin (1:2000, 0.5 μg/mL, Abcam, USA), G‐actin (1:1000, 0.5 μg/mL, Abcam, USA), GAPDH (1:5000, 0.5 μg/mL, Abcam, USA), LATS1 (1:5000, 1 μg/mL, Abcam, USA), p‐LATS1 (Tyr1079, 1:1000, 1 μg/mL, Cell Signaling Technology, USA), YAP (1:1000, 0.5 μg/mL, Cell Signaling Technology, USA), p‐YAP (Ser127, 1:1000, 1 μg/mL, Cell Signaling Technology, USA) and α‐catenin (1:500, 1 μg/mL, BD Transduction Laboratories, USA). Next, the membrane was incubated with the secondary antibody (1:5000, Cell Signaling Technology, USA) for 1 h at room temperature. Thereafter, the membrane was washed three times with TBST, and then the proteins were detected using an imaging system.

### 2.7 Cell immunofluorescence

Endplate chondrocytes were fixed with 4% paraformaldehyde and then were incubated with 0.3% Triton X‐100 solution and 2% sheep serum blocking solution. The primary antibodies YAP (1:100, 0.5 μg/mL, Cell Signaling Technology, USA), ACAN (1:300, 1 μg/mL, Abcam, USA), COL2A1 (1:250, 0.5 μg/mL, Abcam, USA) and α‐catenin (1:800, 1 μg/mL, Cell Signaling Technology, USA) were added and incubated overnight at 4°C. Next, the fluorescent secondary antibody was added, followed by incubation at room temperature for 30 min and DAPI staining for 5 min in the dark. After washing with PBS, the target protein was detected by laser confocal microscopy (Leica, Germany).

### 2.8 Statistical analysis

SPSS 18.0 (USA) software was used for statistical analysis. All the measurement data were tested to conform to a normal distribution. The comparison between two groups of measurement data was analysed by independent sample t‐test. The comparison among three groups of measurement data and above was analysed by ANOVA (one‐way analysis of variance). *P* < 0.05 was deemed to indicate a statistically significant difference.

## 3 RESULTS

### 3.1 Tension can inhibit the phenotypic expression of endplate chondrocytes and change the cell morphology

**FIGURE 1:**
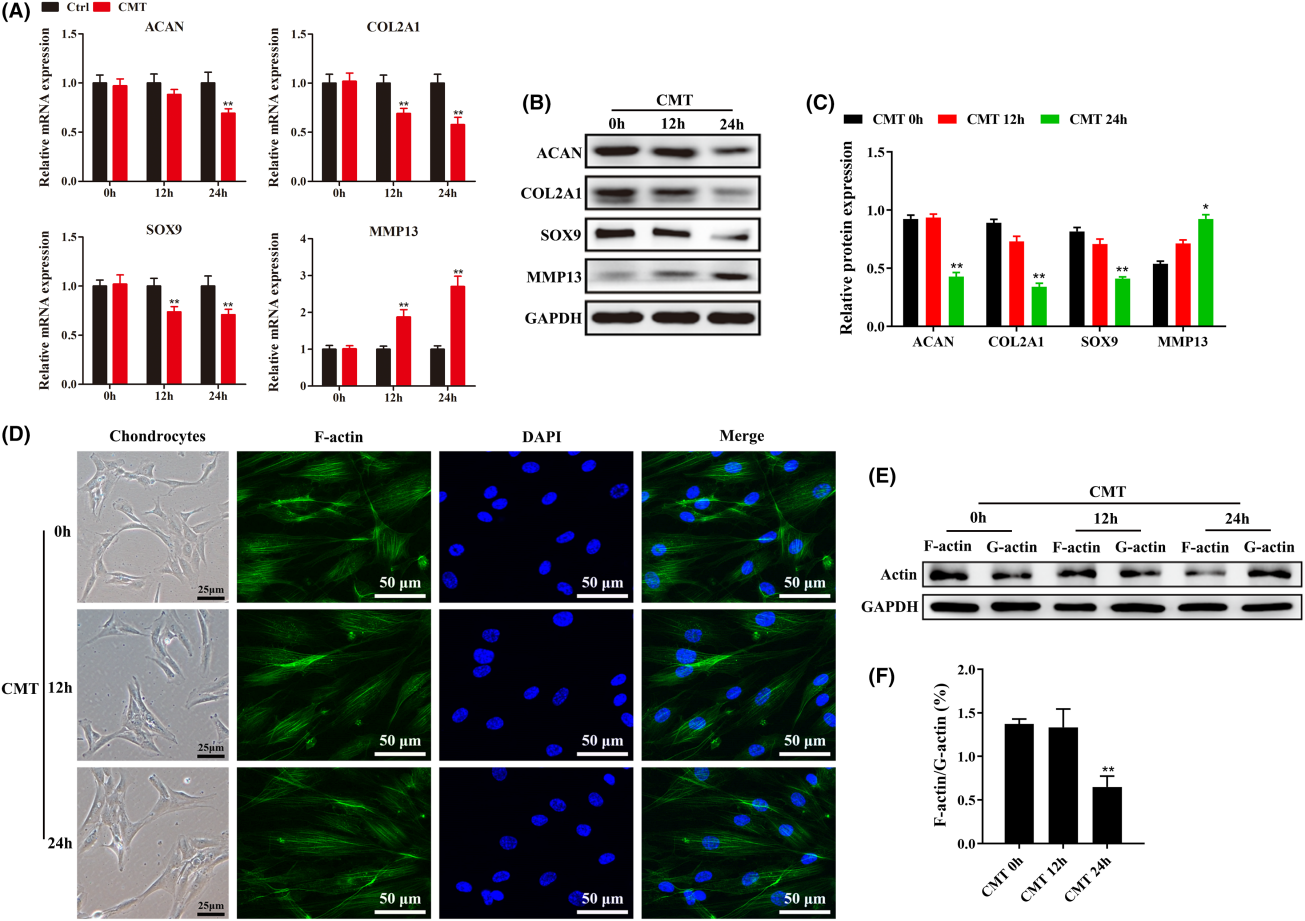
Cyclic mechanical tension (CMT) inhibits the phenotype expression and changes the cell morphology of endplate chondrocytes. (A–C) RT‐qPCR and western blot were used to detect the expression changes of phenotype‐related genes and proteins in endplate chondrocytes before and after tension loading. (D) The changes in actin cytoskeleton of endplate chondrocytes after tension loading were observed by microscope and phalloidin staining. (E,F) Western blot was used to detect the expression level of F‐actin and G‐actin in endplate chondrocytes after tension loading (*n* ≥ 3, **p < 0.01).

The mRNA and protein expression levels of phenotypic markers in endplate chondrocytes (e.g. ACAN, COLII, Sox9) were gradually down‐regulated with prolonged tension loading (*p* < 0.05) (Figure 1A–C). In addition, phalloidin staining showed that the morphology of endplate chondrocytes was polygonal without tension, but endplate chondrocytes showed long spindle‐shaped changes under tension (Figure 1D). The ratio of F‐actin to G‐actin protein expression level decreased significantly with prolonged tension loading (*p* < 0.05) (Figure 1E,F). These findings suggest that tension can induce less ECM production or increase MMP13 and remodel their cytoskeleton.

### 3.2 Under tension, the Hippo signalling pathway in the endplate chondrocyte is significantly activated

**FIGURE 2:**
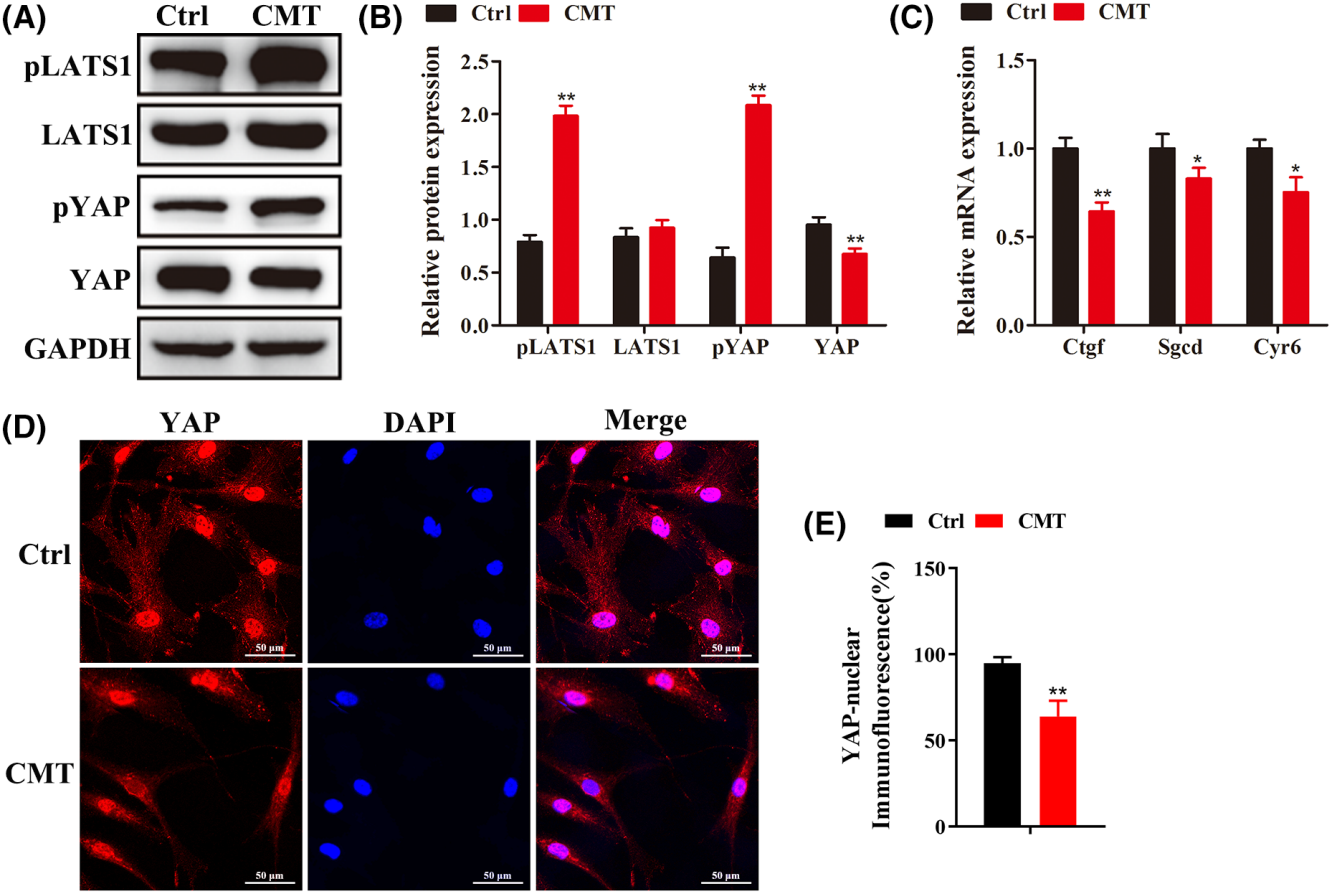
CMT promotes the activation of Hippo signalling pathway in endplate chondrocytes. (A,B) Western blot was used to detect the expression level of Hippo downstream molecules in endplate chondrocytes before and after tension loading. (C) RT‐qPCR was used to detect the expression of YAP target genes before and after tension loading. (D,E) The expression and distribution of YAP protein was detected by immunofluorescence before and after tension loading (*n* ≥ 3, *p < 0.05, **p < 0.01).

Western blotting results showed that the protein content of pLats1 and pYAP increased significantly (*p* < 0.05), the content of YAP decreased, and that of Lats1 did not change significantly under tension (Figure 2A,B). RT‐qPCR results showed that the expression levels of *Ctgf*, *Sgcd* and *Cyr6* downstream target genes of YAP were significantly down‐regulated (*p* < 0.05) (Figure 2C). In addition, immunofluorescence staining revealed a significant reduction in YAP in the nucleus of endplate chondrocytes after tension (*p* < 0.05) (Figure 2D,E).

### 3.3 Overexpression of YAP can alleviate the effect of the inhibition of tension on the phenotypic expression of endplate chondrocytes

**FIGURE 3:**
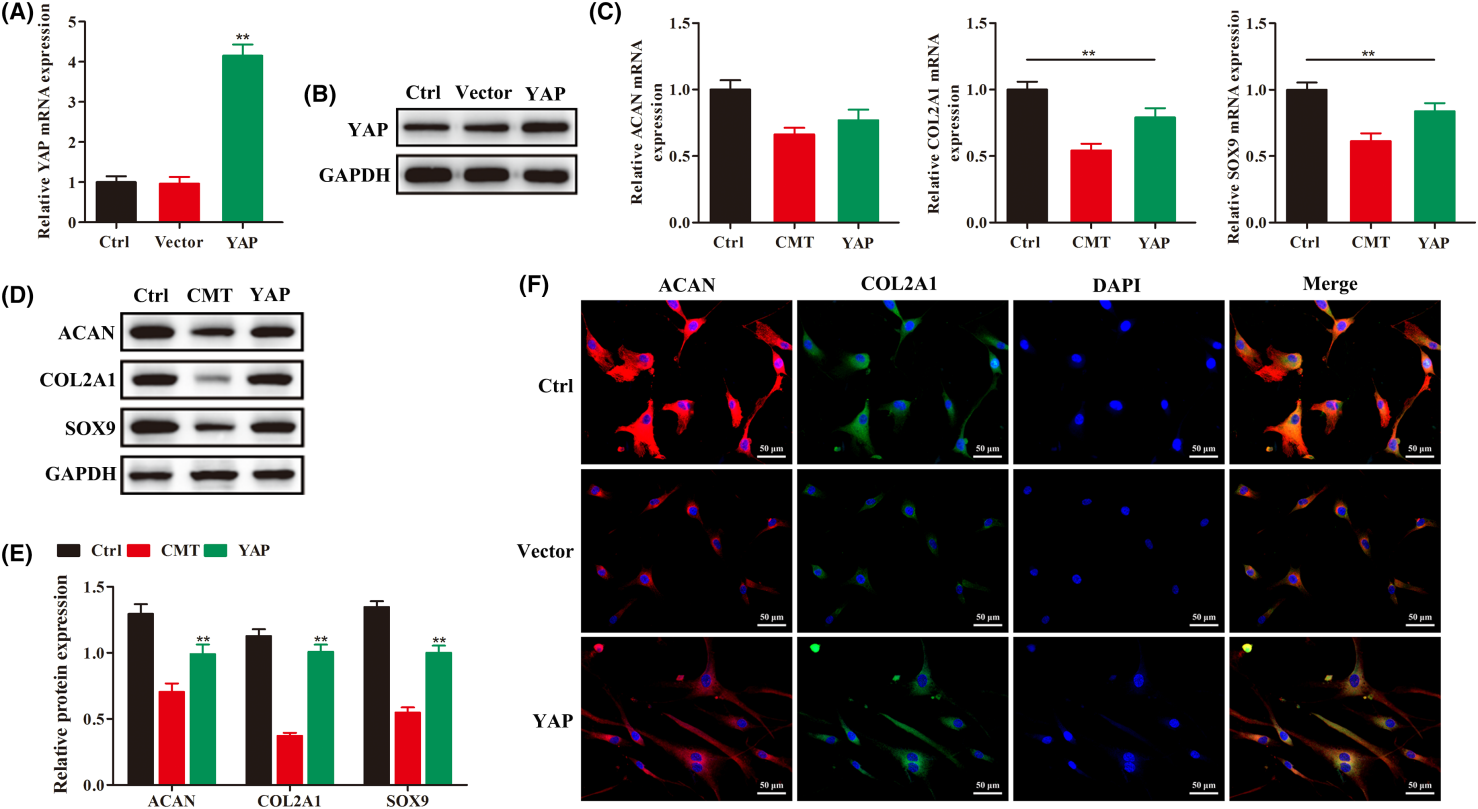
Effect of the regulation of YAP on the phenotype expression of endplate chondrocytes after CMT. (A,B) Detection of the efficiency of YAP overexpression by RT‐qPCR and western blot. (C–F) RT‐qPCR and western blot were used to detect the changes of phenotype‐related genes and proteins in endplate chondrocyte proliferation after YAP overexpression under CMT (*n* ≥ 3, **p < 0.01).

YAP was overexpressed (Figure 3A,B) and then the expression of chondrocyte phenotypic genes and proteins were detected by RT‐qPCR, Western blotting and immunofluorescence. When YAP was overexpressed, and tension was applied again, inhibition of the original endplate chondrocyte phenotype was significantly improved (*p* < 0.05) (Figure 3C–F).

### 3.4 Inhibition of α‐catenin can regulate the cytoskeleton, promote YAP nuclear internalization and maintain chondrocyte phenotypic expression

**FIGURE 4:**
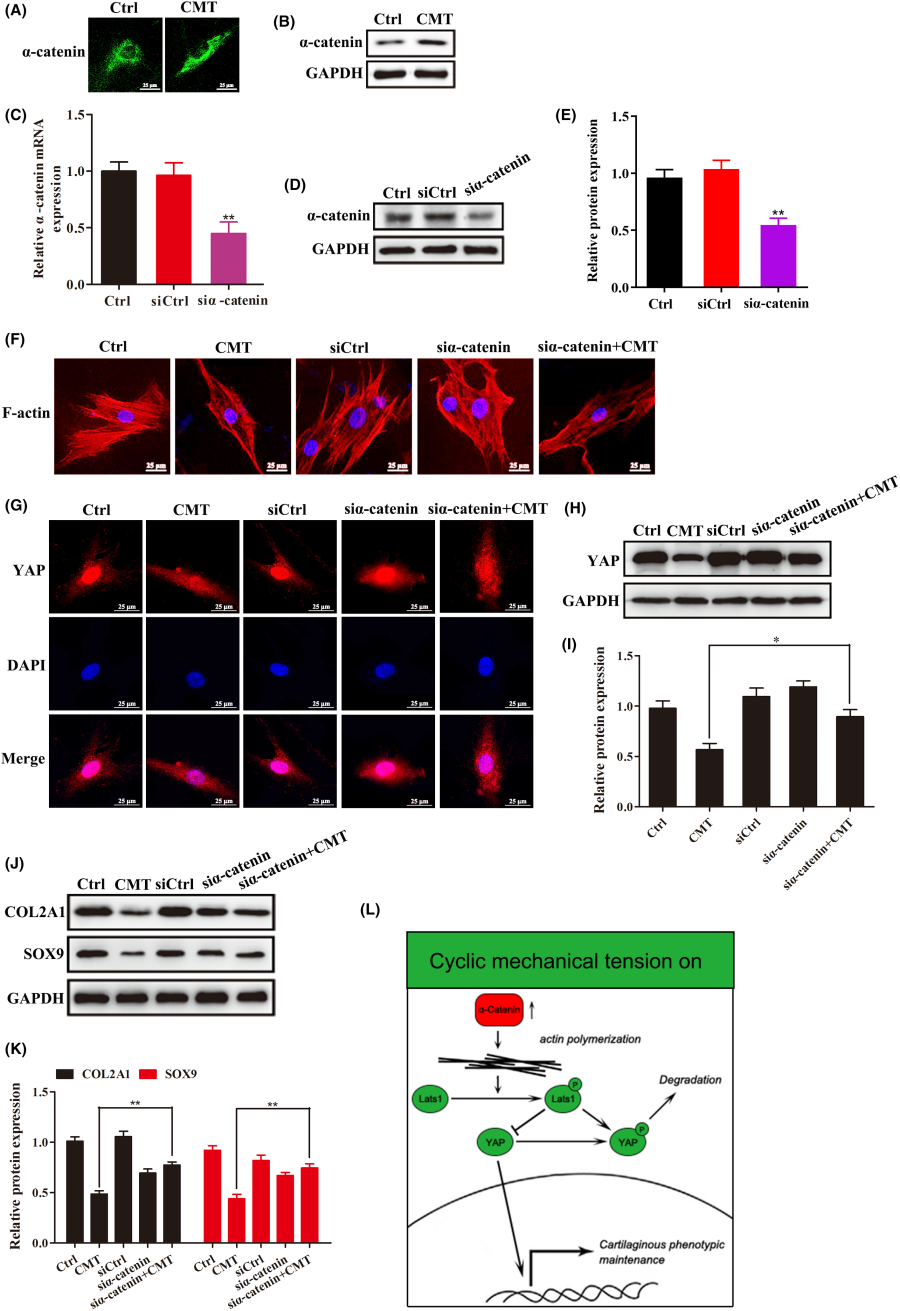
Inhibition of α‐catenin can regulate actin cytoskeleton, promote the YAP nuclear localization and maintain the phenotype expression of chondrocytes. (A,B) Immunofluorescence staining was used to detect the expression level of α‐catenin protein in endplate chondrocytes before and after tension loading. (C–E) Detection of the efficiency of α‐catenin inhibition by RT‐qPCR and western blot. (F) Phalloidin staining was used to detect the changes in actin cytoskeleton of endplate chondrocytes after α‐catenin inhibition under CMT. (G–I) Immunofluorescence staining and western blot were used to detect the expression and distribution of YAP protein in endplate chondrocytes after α‐catenin inhibition under CMT. (J,K) Western blot was used to detect the expression changes of phenotype‐related proteins in endplate chondrocytes after α‐catenin inhibition under CMT. (L) Diagram of the mechanism.

Tension could up‐regulate α‐catenin expression (Figure 4A,B). α‐Catenin demonstrated inhibitory effects on the endplate chondrocyte cytoskeleton, the Hippo signalling pathway and cell phenotype (*p* < 0.05) (Figure 4C–E). Phalloidin staining showed that α‐catenin inhibition could change the skeletal morphology of endplate chondrocytes from polygonal to oval, and the cell morphology showed no obvious spindle deformation after tension (Figure 4F). In addition, the level of YAP intranuclear migration was not reduced by tension (Figure 4G–I). Endplate chondrocyte extracellular matrix secretion was also enhanced (Figure 4J,K).

## 4 DISCUSSION

Endplate chondrocytes are the only cells in the endplate cartilage, and they can sense mechanical forces from tissue structures and respond to external signals by modulating the production of extracellular matrix components.^19^ Tension plays a key role in this process because chondrocytes are embedded in the lacunae of cartilage tissue through surface adhesion, the main effect of which is tension.^6^ Excessive tension conditions lead to reduced proteoglycan synthesis in endplate chondrocytes.^7^, ^20^ In this study, we found that primary cultured human cervical endplate chondrocytes in vitro could significantly inhibit the secretory function of cells after applying cyclic tension, which is consistent with previous study. Under normal conditions, there are two forms of actin in the cell, namely globular actin (G‐actin) dissolved in the cytoplasm in a soluble state and fibrous actin (F‐actin) in an insoluble state. F‐actin is the main structural component of cytoskeleton microfilaments. There is a dynamic balance between the two forms of actin. In this study, we also observed that the tension could ‘stretch’ the skeleton of the endplate chondrocyte, reduce the ratio between F‐actin and G‐actin, enhance the depolymerization of the cell microfilaments and change the cell morphology as a spindle deformation. The conversion between intracellular mechanical and chemical signals involves mechanically induced changes in protein function, but how the above physical and biochemical factors are coupled and how they are further integrated to achieve cellular mechanical sensing remain mysteries to be revealed.

The Hippo signalling pathway comprises a conserved group of kinases, the activation of which leads to cell growth inhibition.^21^ In mammals, membrane protein receptors upstream of the Hippo signalling pathway sense growth inhibitory signals from the extracellular environment, undergo a series of kinase phosphorylation reactions and ultimately act on the downstream effectors YAP and TAZ. YAP and TAZ then interact with cytoskeletal proteins and are trapped in the cytoplasm, unable to enter the nucleus to perform their transcriptional activation functions and regulate organ size and volume.^22^ YAP promotes chondrocyte proliferation but binds different transcription factors to inhibit maturation after chondrocyte differentiation.^23^ Jing et al.^24^ reported that mechanical growth factor (MGF) could activate the RhoA/YAP signalling to protect chondrocytes from mechanical overload injury. Although the above studies suggest that the Hippo pathway actively regulates the intrachondral environment through the YAP pathway, it is unclear whether the Hippo/YAP pathway also plays a corresponding role in the process of tension acting on endplate chondrocytes. In this study, we found that the Hippo signalling pathway was significantly activated under tension, the downstream effector molecule phosphorylated YAP was significantly increased, nuclear YAP was significantly reduced, and the transcription of downstream genes initiated by YAP was reduced. By overexpressing YAP, we observed that inhibition of the endplate chondrocyte phenotype by tension was significantly alleviated. Therefore, we speculate that excessive tension may reduce the nuclear localization of YAP by changing the cytoskeleton, and ultimately weaken its role in maintaining the cartilage phenotype. Therefore, we believe that the Hippo pathway is a key pathway through which tension stimulation acts on endplate chondrocytes.^25^

In most cases, the deformation of mammalian cells is mainly caused by cyclic polymerization of actin and myosin.^26^ To effectively receive tension signals, actin in the cytoskeleton of transmembrane proteins on the cell surface is connected to the extracellular matrix, and the transmembrane proteins themselves have multiple adhesion connections in the cytoplasm in their tails, thus constituting a physical coupling network extracellularly to intracellularly.^27^ Cell functions can also be activated or suppressed by tension, which plays a role in mechanical and chemical signal transduction. Studies have found that increased expression of actin stress fibres can inhibit the differentiation and maturation of chondrocytes while destroying the stress fibres can restore the phenotype of chondrocytes.^28^, ^29^ α‐Catenin is a mechanically sensitive protein in the CCC and an actin‐binding protein, which is necessary for the mechanical coupling of cadherin and actin myosin.^30^ Recent studies have proved that α‐catenin and its actin‐binding protein partners participate in the process of cadherin transmitting mechanical force to the cytoskeleton.^31^, ^32^, ^33^ Previous study approved the involvement of α‐catenin in Hippo pathway activation as a result of cytoskeletal changes.^34^ In this study, we found that the level of α‐catenin protein in endplate chondrocytes was significantly increased under tension. After inhibiting its expression, the morphology of the endplate chondrocytes became oval, and the edges became smoother. In addition, cell spindle deformation induced by the original tension did not appear, and the cell morphology was a flat oval shape. The possible reason is the decrease in α‐catenin protein resulting in impediment of the cell tension signal coupling network and local relaxation of the cytoskeleton. The inhibition of α‐catenin regulates the intranuclear YAP migration level in endplate chondrocytes to resist the negative effect of tension. In addition, phenotypic gene and protein expression of endplate chondrocytes were slightly down‐regulated after α‐catenin inhibition. The original significant down‐regulation was restored after tension loading. Therefore, we conclude that tension causes bidirectional regulation of the phenotypic expression of endplate chondrocytes through the α‐catenin/actin skeleton/YAP pathway. On the one hand, when the above pathways are intact, tension manifests as inhibition of chondrocyte phenotypic expression. On the other hand, when the above pathways are disrupted, the effect of tension on the maintenance of the chondrocyte phenotype is weakened, as well as the ability of chondrocytes to secrete extracellular matrix. Further study on the mechanism actin‐Lats‐YAP pathway induces chondrocyte degeneration is still needed.

## 5 CONCLUSION

The current study preliminarily revealed the transmission pathway of tension signals in endplate chondrocytes and their regulatory effects on the chondrocyte phenotype. Specifically, tension up‐regulates the expression level of α‐catenin in endplate chondrocytes. α‐Catenin activates the Hippo pathway through the actin skeleton, thereby inhibiting the phenotypic expression of endplate chondrocytes (Figure 4L). This mechanism provided a new therapeutic idea to alleviate or reverse endplate cartilage degeneration.

## AUTHOR CONTRIBUTIONS

**Min Zhang:** Formal analysis (equal). **Shouliang Xiong:** Data curation (equal). **Daokuan Gao:** Data curation (equal); investigation (equal). **Chen Liu:** Data curation (equal); investigation (equal). **Liang Xiao:** Conceptualization (equal); writing – original draft (equal); writing – review and editing (equal).

## FUNDING INFORMATION

This study was funded by the National Natural Science Foundation of China (grant no. 82002358).

## CONFLICT OF INTEREST STATEMENT

The authors declare that they have no competing interests.

## CONSENT TO PARTICIPATE

All patients who underwent surgery were aware of this study and provided written informed consent before participating.

## Data Availability

The data that support the findings of this study are available from the corresponding author upon reasonable request.
